# Nurse-Led Approaches to Lowering Alcohol Use among Adolescents: Study Findings

**DOI:** 10.3390/nursrep14020054

**Published:** 2024-03-25

**Authors:** Maria Teresa Moreira, Andreia Lima, Carla Sílvia Fernandes, Ariana Pereira, Dulce Lemos, Lúcia Pereira, Raquel Delgado, Sandra Rodrigues, Francisco Sampaio

**Affiliations:** 1 CINTESIS@RISE, Nursing School of Porto (ESEP), 4200-450 Porto, Portugal; amlima@ufp.edu.pt (A.L.); carlafernandes@esenf.pt (C.S.F.); franciscosampaio@esenf.pt (F.S.); 2 Institute of Research, Innovation and Development Fernando Pessoa Foundation (FP-I3ID, FP-BHS), 4200-253 Porto, Portugal; sandrar@ufp.edu.pt; 3 Escola Superior de Saúde Fernando Pessoa, Rua Delfim Maia, 334, 4200-253 Porto, Portugal; 4 Higher School of Health, Polytechnic Institute of Viana do Castelo, 4900-314 Viana do Castelo, Portugal; 5 PSHC—Portugal Sénior Health Care, 4905-152 Barcelos, Portugal; 6 ULS Alto Ave, 4820-273 Fafe, Portugal; dmmplemos@arsnorte.min-saude.pt (D.L.); lapereira@arsnorte.min-saude.pt (L.P.); 7 IPSS Rua da Quintã, 4820-422 Fafe, Portugal; raquel.roby@acr-fornelos.pt

**Keywords:** alcohol, adolescents, brief interventions, consumption, FRAMES method

## Abstract

This study delves into how motivational sessions and brief interventions impact students’ alcohol consumption, highlighting the vital role of nurses in fostering positive behavioural changes. The study aims to discern the effects of these interventions, starting with a pre-and post-intervention setup involving 62 students from a private school in northern Portugal. The intervention comprised a session delivered by school and mental health nurses, utilizing the motivational intervention and FRAMES method and a poster offering feedback on alcohol consumption scores. The results indicated that females tended to drink for fewer days and engage in less binge drinking than males. Furthermore, the intervention hinted at a reduction in the number of heavy drinking days. This study underscores the importance of including healthcare professionals, particularly nurses, in delivering brief interventions within school settings. The findings carry weight for crafting evidence-based interventions to cultivate healthier adolescent behaviours and enhance overall well-being.

## 1. Introduction

Adolescents are generally advised to refrain from alcohol due to its potential harm to their physical and intellectual development. The ongoing growth of the brain during youth makes it vulnerable to the impact of alcohol. Beyond developmental concerns, alcohol use raises the risk of injuries and accidents, leading to various health problems such as liver damage and addiction. Additionally, alcohol can impair judgment, increasing the likelihood of engaging in risky behaviours, including unprotected sex and driving under its influence, as highlighted by existing studies [1,2]. It is crucial for young individuals to be aware of these risks and make informed choices regarding alcohol, prioritizing their overall well-being.

The World Health Organization (WHO) strongly advocates for the abstinence of alcohol among children [3]. According to WHO [3], “There is no safe level of alcohol consumption for youth, given the detrimental impact of alcohol on their development and heightened vulnerability to harm”. Furthermore, they recommend that governments and other entities enact regulations and initiatives to diminish the availability and accessibility of alcohol to the youth [3]. These measures may involve actions such as elevating the minimum legal drinking age, enforcing restrictions on alcohol advertising, and increasing taxes on alcoholic beverages. It is crucial for young individuals to comprehend the associated risks with alcohol and to make prudent choices concerning its use [4,5].

Evidence shows that alcohol use can negatively impact school performance in youngsters. Alcohol can interfere with gaining knowledge and memory, making it harder for adolescents to memorize and perform appropriately in school. Adolescents who use alcohol are also much more likely to miss school and have lower grades, mainly due to instructional problems and even failure [4,5,6]. Additionally, alcohol use can lead to other issues which could affect school performance, including accidents and injuries, felony problems, and social and family issues [6]. It is vital for youngsters to be aware of the risks related to alcohol use and to make healthful selections on the subject of alcohol to maximize their instructional and private success.

Brief interventions are designed to be concise, with their duration tailored to the specific context and goals. These can include a single session, typically lasting between 5 to 30 min, or multiple sessions, each extending from 20 to 60 min and totaling 2 to 5 sessions. The primary aim is to assist individuals in changing their behavior in a time-efficient manner [7,8,9,10]. Brief interventions are regularly used in the context of substance abuse and dependence. They can effectively assist individuals in reducing their use of alcohol or different materials [10]. Brief interventions can be conducted in various settings, including clinical clinics, colleges, and network organisations [10]. They usually involve an assessment of the man or woman’s substance use, training about the risks and outcomes of substance use, and the improvement of a plan to lessen or forestall substance use. Brief interventions can also include follow-up periods to guide and reveal progress [7,8,9,10].

Group motivational intervention (GMI) represents a therapeutic approach dependent on the principles of motivational interviewing, strategically implemented within a group context. Widely applied to address various behavioural challenges, GMI has demonstrated notable efficacy, particularly in aiding individuals dealing with substance abuse [11]. Within a GMI session, a skilled facilitator or therapist guides participants through discussions and activities aimed at delving into their motivations, aspirations, and obstacles related to behaviour change. The overarching objective is to cultivate a supportive and non-judgmental environment, fostering an atmosphere where participants feel comfortable openly sharing their thoughts, experiences, and concerns [11].

Prevention science has established evidence-based strategies like brief interventions (BI) and motivational interviewing (MI) to address alcohol and substance use among adolescents. One study aimed to analyse the effectiveness of the brief intervention based on motivational interviewing (BIMI) preventive program among 3159 secondary students in a within-subjects design. The results demonstrated that BIMI serves as a preventive strategy, effectively reducing the frequency and quantity of alcohol consumption among adolescents. The study further investigated the impact of elapsed time between sessions on the outcomes, providing valuable insights for prevention and evaluation methodologies [12]. This highlights the significance of incorporating MI-based interventions in adolescent alcohol reduction programs, emphasizing the potential for positive behavioural changes and improved well-being.

FRAMES is an acronym for feedback, responsibility, advice, menu of options, empathy, and self-efficacy. This model is frequently employed in substance abuse treatment and interventions to assist individuals in making positive behavioural changes [13]. The FRAMES model is a structured approach for conducting brief interventions, emphasising fostering a collaborative, non-confrontational relationship with the individual to facilitate change. In essence, it encourages individuals to take ownership of their substance use while exploring a variety of options for reducing or discontinuing their usage. Providers employing this model offer empathy and support, aiding individuals in building self-efficacy, which is the confidence in their ability to instigate change [13].

Furthermore, it is essential to note that evidence suggests that quick interventions using the FRAMES version have been verified as powerful in lowering alcohol use amongst adolescents [13,14]. Numerous research studies have confirmed the efficacy of brief interventions in assisting youngsters to reduce their alcohol consumption and beautifying their standard health and well-being [7,8,9,10]. Nevertheless, it is vital to understand that the effectiveness of these short interventions can range primarily based on numerous elements, consisting of an individual’s motivation to change, the severity of their substance use, and the presence of any occurring mental health disorders [15].

Brief intervention and motivational intervention may be essential tools in healthcare and counselling settings. Depending on the individual’s particular needs and the degree of behaviour, they may provide exclusive benefits. These interventions are often combined to provide a holistic approach to addressing problematic behaviours and promoting positive change [16].

Portuguese adolescents show a high prevalence of substance use, particularly alcohol, with a clear trend of higher consumption among males compared to females [17]. Recognizing that the significance of school-based strategies in prevention and intervention is crucial, Portugal’s educational system supports various activities and services to promote health, prevent diseases, and facilitate early intervention for student health issues [18,19]. These efforts are essential to the Portuguese national health system by enhancing students’ health, well-being, cognitive and emotional development and academic success. Articulated by multidisciplinary teams, including healthcare professionals such as nurses, doctors, psychologists, educational experts, teachers, and educators, these initiatives ensure close collaboration with schools and families to optimize educational outcomes. The extent of school health activities crosses health surveillance, promotion, physical education, disease prevention, health assessments, and early interventions [18,19,20]. Portugal’s affiliation with the Schools for Health in Europe (SHE) Network since 1994 underscores the importance of integrating health and education, promoting physical well-being and learning. Adhering to WHO’s Health Promoting School model, since 2005, schools have been required to incorporate health education into their academic programs [21]. School health services may additionally consist of a school nurse or nurse practitioner. They can care for minor injuries and illnesses, provide statistics, and help with healthy eating, physical activity, and mental health issues. Some schools may additionally have element internships with nearby healthcare providers or clinics that could offer extra assets and aid for students [22]. Providing students with healthcare services is essential to promote their well-being and help them succeed academically in schools [23,24].

The school environment offers great potential for health advertising and the prevention of health problems, which is why schools and health are closely associated. The presence of a school health team, specifically school nurses, is essential for the success of the intervention, as they are trained professionals who can understand and deal with students’ health problems, promote health behaviour, and identify health issues at an early stage. With a multidisciplinary team, it is feasible to ensure that students acquire a comprehensive education that includes health, behavioural education, and well-being [23,24].

Mental health school nurses can be essential in delivering motivational and brief interventions for alcohol use in adolescents. They can provide education, guidance, and support to students and their families. Some results of nurse interventions for alcohol use in adolescents consist of increased awareness and knowledge about the risks of alcohol use, improved communication between them and their families, elevated motivation to change alcohol use behaviour, and reduced alcohol use. Therefore, alcohol screening for risk and harmful intake and brief interventions to prevent the risk of harm and change behaviours are essential resources for nurses in primary health care settings and school health services [25,26].

To address these concerns, this study delves into the effectiveness of one-session brief interventions delivered by nurses in a school setting to reduce or eliminate alcohol consumption among students. This study takes place within the context of the Portuguese adolescent population, recognizing the potential influence of unique cultural and sociodemographic factors on alcohol consumption. Secondly, we underscore the pivotal role of the school health team, especially school nurses and mental health nurses, in delivering motivational and brief interventions, providing a practical perspective within a school environment.

The main goal of this study is to investigate how effective it is when nurses conduct a single session of motivation and brief intervention in a school setting to reduce or eliminate alcohol consumption among students. The study also aims to analyse if there are differences in the effectiveness of the intervention between male and female students.

## 2. Materials and Methods

### 2.1. Study Design

This quantitative, quasi-experimental study uses a pre-test and post-test design (3 months after the intervention) with a single group. To ensure comprehensive, clear, and transparent reporting of our methodology and findings, we structured our manuscript using The Consolidated Standards of Reporting Trials (CONSORT) as a guideline.

### 2.2. Participants

The study was conducted in a private middle and high school in Braga, Portugal. It employed a non-probabilistic and convenience sampling approach to ensure that our participant selection process was robust and reflected the school’s demographics. Participants were selected based on their availability and willingness to engage in the study, considering various factors such as grade levels and classrooms. Specifically, we aimed to include students from different grade levels, including the 9th to the 12th grade, to capture a broad representation of the school’s high school population.

The sample encompassed students of varying ages, gender identities, and academic backgrounds, contributing to a diverse representation in these dimensions. This approach allowed for an assessment of the potential influence of age, gender, and academic factors on the study outcomes. The participants represented the school’s student body, with an equitable distribution of male and female students across different grades. The participation from students with varying grade point averages and academic interests captures a holistic view of the school’s high school students.

The intervention team comprised the school health department, qualified and experienced mental health nurses who deliver motivational and brief interventions and a school nurse. During a designated class, a questionnaire was distributed to the students. They were allocated up to 45 min to fill it out. The data collection process was carefully administered to ensure accuracy and reliability.

### 2.3. Inclusion and Exclusion Criteria

The inclusion criteria for the present study were being a high school student at the school in question. Exclusion from the study occurred if the participant did not meet the inclusion criterion of being a high school student at the school in question and needed a signed informed consent form validated by their legal guardian on the day of data collection. Additionally, refusal by the student to participate also resulted in exclusion from the study.

### 2.4. Measurements

The questionnaire used to assess the alcohol consumption of adolescents was divided into two groups. Part 1 was a sociodemographic profile, and part 2 was a Youth Risk Behavior Survey (YRBS) questionnaire. The socio-demographic profile was used to characterize the sample, encompassing age, gender, scientific area of study, cohabitation, academic/literary qualifications, parents’ profession, and parents’ marital status.

The instrument contains 78 health-related behaviour (HRB) items, composed of multiple-choice questions. Response options are dichotomous (10 items) or ordinal polytomous (66 items) that express the frequency of HRB in different time frames (previous day, past seven days, past 30 days, past 12 months, or during life). In addition to these items, YRBS has two open questions about the respondent’s weight and height. Items are distributed over the following 11 domains: personal safety (ten items); violence (five items); suicide (four items); tobacco use (eight items); alcohol consumption (three items); cannabis use (three items); other drug use (ten items); sexual activity (fifteen items); body weight (eight items); feeding (seven items); and physical activity (five items). According to the original study, the YRBS demonstrates acceptable reliability and validity [27]. In this study, three of those categories, aligning with the research objectives, were evaluated. Santos and Silva validated this self-administered tool for the Portuguese population, showing an average reliability test result of 68.6% by the Kappa index [28].

### 2.5. Intervention

The intervention was administered by two highly trained and qualified mental health nurses who implemented a 45 min structured motivational group intervention. This intervention followed the format of a brief intervention and was based on the FRAMES method, a well-established evidence-based technique known for fostering dialogues that facilitate behavioural change. During the intervention session, a strategically placed poster played a significant role. This poster served both as a visual stimulus and an informative guide. It presented the alcohol consumption scores of students from that academic year, allowing the participants to compare their consumption rates with their peers. This comparative display was instrumental in making the students aware of both commendable and concerning aspects of their alcohol consumption patterns, highlighting the potential consequences of their choices.

Beyond presenting statistical data, the poster provided practical guidance on modifying consumption behaviour. It addressed the complexities and challenges of reducing alcohol consumption and offered insights on overcoming these obstacles to enhance overall well-being. A central theme of the intervention was the belief that positive transformation is attainable. The mental health nurses inspired students to be confident in making positive life changes. This belief, coupled with determination and the necessary support structures, could empower students to reduce or eliminate alcohol intake.

By combining the expertise of the mental health nurses, the FRAMES method standards, and the poster’s visual effect, the intervention aimed to create a nurturing and empowering atmosphere for adolescents. The main goal was to encourage self-reflection, provide essential statistics, and motivate students to embrace healthier choices concerning alcohol consumption.

### 2.6. Outcome Measures

To compare the outcomes of the motivational intervention and the intervention on students’ attitudes, perceptions, and behaviours related to alcohol intake, we used a two-pronged assessment strategy. First, a questionnaire was sent via Google Forms, before the intervention, for a baseline assessment of the student’s existing information, attitudes, and health behaviours. It included frequency of alcohol consumption, motivation to pay attention to health risks, and perceptions of drinking norms amongst friends. A follow-up questionnaire was sent three months after the intervention, to assess the continued impact and ability of long-term period adjustments in college students’ perspectives and behaviours related to alcohol after the intervention. This evaluation re-examined the critical areas from the initial questionnaire and additionally supplied insights into behavioural changes, motivational shifts, and a better understanding of consumption attitudes and health risks. Through this twin assessment method, we aimed to gain holistic information on the intervention’s effectiveness in selling healthier attitudes and behaviours among students.

### 2.7. Data Analysis and Storage

Statistical analyses were conducted using the Statistical Package for the Social Sciences (IBM SPSS), version 27.0. The characteristics of the sample were summarized by frequency and percentages. The chi-squared test for association was used to test whether two categorical variables are associated. Every time the assumptions for the chi-squared test were not met, Fisher’s exact test was used. Furthermore, ordinal logistic regression was used to predict an ordinal dependent variable given one or more independent variables. More specifically, it was used to determine which independent variables have a statistically significant effect on the dependent variable and how well the ordinal logistic regression model predicts the dependent variable. The level for statistical significance was set at *p* < 0.05 (two-tailed).

Ensuring participant data’s security and confidentiality was paramount throughout the study. All collected data were securely stored in electronic databases with restricted access to authorized personnel only. Personal identifiers were anonymized or coded to protect the privacy of the participants.

Moreover, a backup system was implemented to prevent data loss, and regular checks were conducted to verify the integrity of stored information. The research team adhered to ethical guidelines and regulations concerning data management, guaranteeing the responsible and ethical handling of sensitive participant information.

### 2.8. Ethical Considerations

To ensure respect for ethical principles in conducting this study with adolescents, international ethical guidelines were followed, including obtaining informed consent from both the adolescents and their legal guardians, ensuring the confidentiality and privacy of the participants, minimizing risks and discomfort, involving the adolescents in the research process, and providing appropriate support and resources. These principles were respected at all stages of the study, from data collection to analysis and interpretation of the results, to ensure validity, reliability, and respect for the rights and dignity of the participants. Participants were informed of the study objectives, and those who did not provide parental consent or did not complete the questionnaires were excluded. The Declaration of Helsinki conducted the study, and the protocol was approved by the Ethics Committee of the University of Fernando Pessoa (Approval Number: ESS/PI—205/21). The information on the research project can be found at https://osf.io/zgrvt/, accessed on 10 March 2024.

## 3. Results

The sample comprised 62 participants: 27 (43.55%) females and 35 (56.45%) males between the 9th and 12th grades. Table 1 presents more detailed characteristics of the sample.

### Findings from the Prospective Study

The effectiveness of the reduction in alcohol consumption was assessed by comparing baseline and follow-up measurements. The results did not show statistically significant associations between both moments of intervention and gender, in relation to alcohol use among the participants (Table 2). The respondents that never drank alcohol were not included in the analysis after the first question. The question of how old the participants were when they drank alcohol for the first time was not included in the follow-up, considering the age remained the same.

A cumulative odds ordinal logistic regression with proportional odds was run to determine the effect of gender and intervention on the number of days the participants drank at least one alcoholic drink. The assumption of proportional odds was met, as assessed by a full likelihood ratio test comparing the fit of the proportional odds model to a model with varying location parameters, χ^2^_(10)_ = 11.65, *p* = 0.309. The deviance goodness-of-fit test indicated that the model fit the observed data well, χ^2^_(16)_ = 12.08, *p* = 0.739. The final model statistically significantly predicted the dependent variable over and above the intercept-only model, χ^2^_(2)_ = 6.52, *p* = 0.038. The odds of being in a lower category of the dependent variable for females was 0.33 (95% CI, 0.13 to 0.81) times that for male subjects, χ^2^_(1)_ = 5.90, *p* = 0.015 with a statistically significant effect. At the same time, intervention was not statistically significant (*p* > 0.05).

Furthermore, a cumulative odds ordinal logistic regression with proportional odds was run to determine the effect of gender and intervention on the number of heavy drinking days. The assumption of proportional odds was met, as assessed by a full likelihood ratio test comparing the fit of the proportional odds model to a model with varying location parameters, χ^2^_(10)_ = 13.53, *p* = 0.195. The deviance goodness-of-fit test indicated that the model fit the observed data well, χ^2^_(16)_ = 15.21, *p* = 0.509. The final model statistically significantly predicted the dependent variable over and above the intercept-only model, χ^2^_(2)_ = 13.46, *p* = 0.001. The odds of being in a lower category of the dependent variable for females was 0.20 (95% CI, 0.06 to 0.63) times that for male subjects, χ^2^_(1)_ = 7.46, *p* = 0.006 with statistically significant effect. The odds of being in a higher category of the dependent variable for pre-intervention was 0.30 (95% CI, 0.11 to 0.84) times that for post-intervention, χ^2^_(1)_ = 5.32, *p* = 0.021 with statistically significant effect. The regression model for drinking in school spaces was not significant.

## 4. Discussion

This intervention was a collaborative trial involving a healthcare team composed of multiple health professionals, including mental health nurses, school nurses, nutritionists, and psychologists. Nurses played a significant role in delivering these quick interventions to students on the faculty premises. In addition to assessing alcohol use with the YRBS, we also considered nursing diagnoses as outcomes, highlighting the critical role of nurses in identifying and addressing adolescent health problems. Overall, the study aimed to contribute to developing effective, evidence-based interventions delivered primarily by healthcare professionals, particularly nurses, to inspire children to adopt healthier behaviours and improve their typical well-being and to market options that prioritize health over consuming alcohol.

In a school context, motivational and rapid interventions have proven to be powerful tools for inspiring excellent behavioural changes among students, especially those with unstable behaviours, including alcohol consumption [2,29]. The FRAMES technique was implemented during designated class sessions, where students were provided with the YRBS questionnaire. Following the completion of the questionnaire, nurses utilized the FRAMES approach to initiate one-on-one or group discussions with students based on their reported alcohol consumption patterns. Visual aids, such as posters, were employed to reinforce key messages and provide a tangible reference for students [13,14]. Overall, the FRAMES technique was integrated into the intervention to facilitate meaningful and personalized conversations with students, promoting self-reflection, and empowering them to make informed choices regarding their alcohol consumption. The combination of personalized feedback, responsibility, advice, a menu of options, empathy, and a focus on self-efficacy contributed to the effectiveness of the nurse-led interventions in the school setting.

School health interventions are of particular importance because children spend a considerable part of their adolescence in this environment, which makes it an ideal measure for preventive measures against volatile behaviour along with alcohol consumption. Alcohol intake with the help of teenagers increases justified problems as it has significant negative consequences for both physical and intellectual fitness [30,31,32].

One immediate concern with alcohol intake during early life is its mind-enhancing impact, which can cause fatal problems related to memory, attention, and learning. Excessive alcohol consumption can also lead to unstable behaviour, including drunk driving, unprotected sex, or engaging in physical altercations [31]. In addition, frequent alcohol consumption during early life may increase the risk of developing alcohol dependence in adulthood and lead to long-term physical health problems, including liver, pancreatic, and coronary heart disease [31,32].

Alcohol consumption throughout the early years is a complicated behaviour, influenced by various individual, social, and cultural elements. Peer stress and the desire to fit into social enterprises are usually the number one social influence that make adolescents experiment with alcohol [33]. Adolescents are deeply encouraged by their social environment, primarily by friends and classmates. The stress of trying alcohol can be overwhelming, and many children may also feel pressure to conform to avoid exclusion or rejection. Therefore, educational efforts regarding alcohol consumption at some point in early life should consist of techniques for coping with peer tension and addressing the desire for belonging within a social organization. Adolescents need to learn that they can choose not to consume alcohol while still maintaining active social interactions with their peers [33].

Understanding the significant impact of alcohol consumption is crucial, as it affects not only physical health but also the intellectual and emotional development of adolescents. Excessive drinking can precipitate risky behaviors, leading to physical injuries, challenges in academic settings, and social difficulties. Moreover, it can detrimentally affect learning outcomes and compromise the future prospects of young individuals [34].

Several studies, including systematic reviews and meta-analyses, have tested the effectiveness of brief interventions in reducing youth alcohol intake [35,36,37,38]. Effects consistently suggest that rapid interventions are effective in reducing alcohol intake and related problems, with statistically significant reductions in fluid intake consistent with week and frequency of consumption, specifically in adolescents who engage in risky drinking behaviours [35,36,37,38].

These findings are consistent with previous studies on motivational and rapid interventions in the context of alcohol consumption in young people [31,39,40,41]. These interventions have proven effective in various settings, including schools, primary care, and emergency departments, in reducing alcohol intake and related problems [2,40,41,42].

Gender-based differences in alcohol consumption among students are a well-documented phenomenon in various studies on adolescent behaviour [43]. The present study also observed differences in drinking patterns between male and female students. Historically, research has indicated that male students tend to consume alcohol at higher rates compared to their female counterparts [43,44]. This pattern can be attributed to several factors, including societal norms, peer influences, and differences in risk perception. In our study, we found that male students, on average, reported higher levels of alcohol consumption and a greater frequency of risky drinking behaviours when compared to female students. This difference highlights the importance of gender-specific interventions and educational programs tailored to address the specific needs and challenges faced by male and female students concerning alcohol use [43,44,45].

In conclusion, the findings highlight the important role that nurse-led interventions can play in reducing alcohol use among adolescents. Nurse-led interventions benefit from the expertise and training of healthcare professionals. Nurses are well-equipped to provide accurate information about the risks associated with alcohol use, both in the short and long term. Their guidance empowers young individuals to make informed decisions about their alcohol consumption. Often employed by mental health nurses, MI techniques create a non-confrontational and supportive environment for students. By engaging in open and empathetic conversations, nurses help students explore their motivations, goals, and challenges related to alcohol use. This approach enhances motivation for change.

While this study exhibits potential, it is crucial to acknowledge its limitations. Firstly, the reliance on self-reported data introduces potential biases, including social desirability and recall biases, which may compromise the accuracy of reported alcohol consumption. The absence of a control group presents challenges in establishing definite causality, making it difficult to isolate the effects of nurse-led interventions. Recognizing the potential for selection bias and confounding factors is essential for a nuanced interpretation of the study’s outcomes.

Additionally, the relatively short duration of the intervention raises concerns about capturing the long-term effects and potential relapse of drinking behaviour within the three-month follow-up period. The study, conducted in a specific private school in northern Portugal, may limit the generalizability of findings to a broader adolescent population. The small sample size of 62 students, despite efforts to ensure diversity, warrants caution when extrapolating results to adolescents in different settings or cultural contexts, impacting the external validity of the findings.

The reliance on self-reported data introduces the possibility of social desirability bias and recall biases, potentially impacting the accuracy of outcome measures. Moreover, the chosen measures may not fully capture the diverse spectrum of adolescent drinking behaviours.

Resource limitations, including time constraints within the school setting and potential variations in the availability of qualified personnel, may have influenced the depth and thoroughness of interventions, impacting overall study outcomes.

To address these limitations, future research should conduct a more comprehensive investigation into the broader impact of nurse-led interventions, considering extended follow-up periods. Additionally, exploring the complex mechanisms underpinning the effectiveness of such interventions over time will contribute to a deeper understanding of their sustained impact.

## 5. Conclusions

The findings of this study provide valuable insights into adolescent alcohol consumption patterns, indicating that the sample had initial exposure to alcohol at the age of 13 or older. Notably, female participants exhibited a positive trend towards reduced drinking days and fewer instances of binge drinking, while the intervention suggested a potential decrease in heavy drinking days.

These results emphasize the potential effectiveness of motivational and brief interventions delivered by nurses in preventing and mitigating risky alcohol behaviours among adolescents. The study underscores the importance of early intervention in effectively communicating the risks of alcohol use and fostering behavioural receptiveness. School nurses are encouraged to integrate motivational brief interventions into their practice, addressing not only the physical risks but also the social and emotional factors influencing adolescent behaviour.

The role of school nurses is pivotal in facilitating positive behavioural changes related to alcohol consumption through counselling and guidance. Engaging adolescents in discussions about self-esteem, self-image, and responsible decision-making can significantly contribute to their intellectual and emotional well-being. Providing accurate information about alcohol, promoting awareness, and instilling a sense of accountability for choices made are essential components of effective intervention.

Beyond its immediate findings, this research contributes valuable insights into the effectiveness of nurse-based interventions in the Portuguese context. The study highlights the potential of brief interventions, particularly when delivered by healthcare professionals like mental health nurses in a school setting, to influence health behaviours, specifically addressing binge drinking. The crucial role of school nurses in promoting positive behavioural changes is evident, emphasizing the potential of evidence-based interventions to enhance the well-being of adolescents. Practitioners are encouraged to integrate such interventions into their clinical practice to address alcohol-related issues effectively. Future efforts should focus on refining and expanding these interventions to ensure sustained impact and relevance in diverse contexts.

## Figures and Tables

**Table 1:** Participants’ sociodemographic characteristics (*n* = 62).

	*n* (%)
Age	
15	32 (51.61)
16	13 (20.97)
17	13 (20.97)
≥18	4 (6.45)
Parents’ marital status	
Married/cohabiting	46 (74.19)
Divorced	9 (14.52)
Other	7 (11.29)
Household	
Mother and father	46 (74.19)
Mother or father	7 (11.29)
Other family members	2 (3.23)
Other situation	7 (11.29)
Self-perceived school performance	
Insufficient	3 (4.84)
Sufficient	10 (16.13)
Good	32 (51.61)
Very good	17 (27.42)
Self-perceived health status	
Bad	1 (1.61)
Good	13 (20.97)
Very good	34 (54.84)
Excellent	14 (22.58)

**Table 2:** Participants’ self-rated alcohol use in the intervention group at baseline and follow-up, divided by gender. The number of participants is followed by the respective percentage *n* (%).

		Baseline			*p*-Value Gender	Follow-Up			*p*-Value Gender	*p*-Value Intervention
Question 1	Never 8–12 years old ≥13	Total *n* = 62	Female *n* = 27	Male *n* = 35	0.319 ^a^ (n.s.)		---	---	---	---
		25 (40.30)	13 (48.2)	12 (34.4)						
		12 (19.40)	6 (22.2)	6 (17.1)						
		25 (40.30)	8 (29.6)	17 (48.6)						
Question 2	0 days 1 to 5 days ≥6 days	Total *n* = 37	Female *n* = 14	Male *n* = 23	0.395 ^a^ (n.s.)	Total *n* = 29	Female *n* = 11	Male *n* = 18	0.361 ^a^ (n.s.)	0.879 ^a^ (n.s.)
		13 (35.10)	6 (42.90)	7 (30.4)		9 (31.00)	5 (45.50)	4 (22.20)		
		11 (29.70)	5 (35.7)	6 (26.10)		8 (27.60)	3 (27.30)	5 (27.80)		
		13 (35.10)	3 (21.4)	10 (43.5)		12 (41,40)	3 (27.30)	9 (50.00)		
Question 3	0 days 1 to 5 days ≥6 days	Total *n* = 37	Female *n* = 14	Male *n* = 23	0.382 ^a^ (n.s)	Total *n* = 29	Female *n* = 11	Male *n* = 18	0.098 ^a^ (n.s.)	0.200 ^a^ (n.s.)
		27 (73.00)	12 (85.7)	15 (65.2)		15 (51.70)	8 (72.70)	7 (38.9)		
		6 (16.20)	1 (7.1)	5 (21.7)		9 (31.00)	3 (27.3)	6 (33.3)		
		4 (10.80)	1 (7.1)	3 (13.0)		5 (17.20)	0 (0.00)	5 (27.8)		
Question 4	0 days ≥1 day	Total *n* = 37	Female *n* = 14	Male *n* = 23	0.407 ^b^ (n.s.)	Total *n* = 37	Female *n* = 14	Male *n* = 23	0.407 ^b^ (n.s.)	0.124 ^b^ (n.s.)
		31 (83.80)	11 (78.6)	20 (87.0)		28 (96.60)	11 (78.60)	20 (87.0)		
		6 (16.20)	3 (21.4)	3 (13.0)		1 (3.40)	3 (21.4)	3 (13.0)		

^a^ Chi-squared test. ^b^ Fisher’s exact test; Question 1: How old were you when you drank alcohol for the first time, besides sips? (if never please do not answer the following questions). Question 2: In the last 30 days, how many days did you drink at least one alcoholic drink? Question 3: In the last 30 days, how many days did you drink five or more alcoholic drinks in a row, i.e., without a space of 4 h between them? Question 4: In the last 30 days, how many days did you drink at least one alcoholic drink in spaces belonging to the school?

## Data Availability

The data supporting this study’s findings are available from the corresponding author, MTM, upon reasonable request.

## Guidelines and Standards Statement

This research was conducted according to The Consolidated Standards of Reporting Trials (CONSORT) guidelines and standards.
